# Business of medicine

**DOI:** 10.2349/biij.6.2.e14

**Published:** 2010-04-01

**Authors:** BJJ Abdullah

**Affiliations:** Department of Biomedical Imaging, University of Malaya, Kuala Lumpur, Malaysia

**Keywords:** Business, medicine, perspectives

As has often been said, people get set in their ways of thinking, living and believing until they are unable to see situations in ways other than what they are used to. Their brains just refuse to change its paradigm of thinking.

For me, I have always thought of business in a certain way. Firstly, I was never really exposed to the concept of business, going as far back as I can remember. My father always reminded me that you should devote yourself to helping others for the overall good of society. So it should not come as a surprise that all my siblings work in the public service. When I started to work in the public sector serving a tertiary university hospital, the concept of pushing oneself for money or profit was considered “dirty”, even to the point of viewing such individuals as lower life forms!

But over the last several years, while managing a department and pursuing an MBA, one question has been popping up in my mind: “What business are doctors in?” Is it about treating patients to help them recover from whatever ails them, no matter the cost to the society at large? Or is it about providing a patient a limb prosthesis even though he or she cannot afford to pay for it? What about keeping the public healthy first so they do not get sick, and saving all that money and technology? Are we not supposed to provide parking bays for our patients so they will not be driving around in circles while we literally drive into our offices?

Is it about protecting one's discipline against the onslaught of the invaders? Is it about living in our little silos which, incidentally, were artificially created for an era long gone by? Is it about the need to ensure the maximum amount of resources no matter how it is used? Is it about being the first to get all the fancy high-tech gadgets and equipment? Is it about meeting all the performance targets set by the hospital or health authority? Should the insurance industry set guidelines for patients who need treatment? Should the vendors of imaging equipment and drugs be allowed to promote their products only with limited indications? Are they also acting ethically when they lobby the governments to pay for some of these new choices?

If all these questions have not given you a headache yet, here's more: should money be spent on a 3T MRI or on treating patients with expensive chemotherapies where the survival prognosis is only 3- 6 months? Instead, might it be better to purchase ventilators for the paediatric ICU, or a new image-guided or robotic surgical system for the surgeon? Would the money be better invested in implementing a wellness programme run by the primary care doctors? Perhaps the money should be spent on none of the above, but instead on upgrading the car park, the lift system or refurbishing the outpatient clinics.

Choices! Decisions! Discussions, lobbying, politicking, and invariably gridlock, lead to lots of heartache, anger, confusion, disappointment and, eventually, withdrawal and resignation. Unfortunately, throughout our entire training as healthcare professionals, we have never been exposed to this reality of limited resources, competing needs and, more importantly, different value systems. We find that those who shout the loudest, are the most unreasonable and play “politics” are usually the ones who get their way [1]. This then becomes a way of life and the unwritten rule for getting things done.

To answer the question “What business are we in?”, it depends on which view you coming from.

The patients’ perspective of the medical services depends on their view of medicine. If they hold to a paternalistic view of the doctor, then they will be subservient to their doctors, putting up little or no fuss. On the other hand, those who view themselves as customers feel that doctors are at their beck and call to sort out their problems immediately even if it means that their expectations are unrealistic. These patients are not to blame since the hospitals themselves are increasingly behaving like any other commercial service provider, with key performance indicators, visions, missions, etc. The problem may be that even though medicine has a lot of science behind it, is still very much an art. Unfortunately, in the medical management of patients, their care is not similar to having your car serviced or getting your food in four minutes. Despite all the challenges, both groups of patients expect to be told the truth of their conditions and prefer medications that they only have to take once a day to get rapid action, good effect, and few adverse events (common or rare).

Above all else, what most patients and citizens want is the security of knowing that these health services will be there when they need them, that their views and preferences will be taken into account by health professionals, that they will be given the support they need to help themselves, that they can access reliable information about their condition and the treatment options, and that they won't have to worry about the financial consequences of being ill. They also want to be sure that these benefits are equitably delivered and that public resources are being used efficiently for the good of all. Social solidarity and trust will continue to be the essential underpinnings of a sustainable health system [2].

When we look at students who enter the medical discipline, we get a different perspective. Many students who are determined to pursue medicine do so partly because they see a future in which they will work for themselves and control their professional lives in a way unlike their peers in other careers. Even though that vision was a reality generations ago, today it is a seldom-realised fantasy but one that I have to confess is still a widely-held view. In reality, doctors who work in solo practice are increasingly becoming part of a larger network of healthcare plans or some consortium of sorts. The vast majority are either in private groups or employees of medical schools or large networks. Despite all these changes, doctors adamantly protect independence in their relationships with patients and in their other day-to-day activities. Interestingly, most do not enter the profession with the interest in, or equipped with the skills for, administrative responsibilities. It would probably be true to say that we have pre-programmed ourselves with the desire not to run an organisation. I guess there is an inherent rebellious steak that will go against any loss of control or any desire for uniformity or the regimentation that comes as a part of the corporate culture [3].

If you are a practising clinician in the public service, your perspective is probably that ALL resources must be made available for your needs; be they expensive antibiotics which are currently not in the formulary, or more ICU beds to cater for the sick patients, so that they can provide the best for their patients. However, money should not be wasted on making the place comfortable, for example repainting the premises, or upgrading the cafeteria. Public health clinicians have repeatedly re-enforced in their practice that everything that an institution does should be in the patients’ long-term interest.

This raises the question: is there a potential conflict of interest when doctors make decisions in the interests of the organisation as well as that of their patient? The biggest conflict lies in rationing the finite available resources, especially when the parties in the scramble cannot see the needs of others. For example, should USD 2 million be spent on preventing or treating TB cases, versus buying a 3T MRI? What about needs outside the healthcare industry, such as social needs including the need to educate children or invest in road safety and transportation? In reality, medical specialties, as well as technological and procedural services are often overvalued [4].

For those in academia, the challenges are even more dramatic. There is a vital need for freedom of expression and free scientific exchange among the faculty, trainees, and students. They need to have their research and lectures on patho-physiology of health and disease, new diagnostic tools and treatments published widely, while striving to continually upgrade the practice and delivery of health care itself, which for most, is the core of their professional lives. These core values often require challenging the leaders in universities who wish to push a different agenda with the use of key performance indicators, 360^0^ appraisal, grantsmanship and turf battles. However, there are academics for whom these challenges are not entirely relevant, as they feel that academia should not only be about lecturing and publishing. They believe that members of the academia should get out of their ivory towers and become advocates for scientists, engineers, or health care workers who have been unjustly imprisoned or threatened with imprisonment [5]. The responsibility of academics should not be merely be generating and exchanging new knowledge, but applying that knowledge to make a difference in the world so that they fulfil their work’s true potential. One such example is the Duke Global Health Institute which brings together interdisciplinary teams from schools and departments throughout the University to work with partners to solve highly complex health problems and train the next generation of global health scholars [6].

For the managers, their challenges are different. As a consequence of the Reagan and Thatcher eras, as well as the “globalists”, the last several decades has seen a distinct trend where even public institutions are being encouraged to deliver on their objectives. Hospitals, medical institutions and universities also have not been spared. In the United Kingdom there are now direct or indirect financial incentives for medical practitioners who have been given the responsibility of managing funds. Even though the link between practitioners' income and services delivered currently remains weak, financial rewards are based on performance at the level of the individual practice or trust [7]. The increased accountability for the resources used, combined with measurable performance indicators, has added a complexity to the managers' responsibility.

Then there are those who are in the business of generating revenue for their investments. They speak of the need for a good business model with concepts like value proposition (a description of the customer’s problem, the product that addresses the problem, and the value of the product from the customer's perspective), market segment (the group of customers to target, recognising that different market segments have different needs), value chain structure (the firm's position and activities in the value chain and how the firm will capture part of the value that it creates in the chain), revenue generation and margins (how revenue is generated, the cost structure, and target profit margins), position in value network (identification of competitors, complementors, and any network effects that can be used to deliver more value to the customer) and the competitive strategy (how the company will attempt to develop a sustainable competitive advantage, for example, by means of a cost, differentiation, or niche strategy). These “investors” see the provision of healthcare based on business principles as being different from any other commercial business. Therefore, it is now very common to find jargon like mission, vision, customers, key performance indicators, and return on investments being thrown around.

"Customers, not patients” is now the common cry everywhere! So what is a customer? In business-speak, a customer is someone who makes or influences the decision to buy services, regulate business, or affect brand perception. In health care, customers are not just your patients but also include referring medical professionals, families, hospitals, the government, disease and patient advocacy groups, and joint-venture partners. The services we provide must meet their expectations about healthcare, which have changed dramatically in recent decades. People now refer to a rise of "concierge" medical services, in an attempt to beat the competition. While patients are now given designated car parks, free refreshments, and a comfortable environment akin to that of a posh hotel, they also have increased expectations of the care provided to them. Patients who are looking for one-stop shopping (they would rather have their MRI performed and interpreted on the same day as their neurologic/mammographic/cardiologic consultation, rather than having to leave the consulting doctor’s office, schedule an appointment, have the scan, and wait a few more days for results) create a business environment that seriously challenges the traditional territory of diagnostic radiology.

What usually gets lost in the discussion comparing medicine with other services is the fact that you cannot take your body back if the treatment or surgery did not produce the results you asked for; something that you can do with your car or your refrigerator. The difference between the medical trade and other commercial trades is that healthcare deals with acute life-threatening issues, while the mechanic's endeavours do not affect your life immediately.

The increase in global trade raises important new challenges for health and the provision of health-related goods and services. Multinational corporations in the new millennium are increasingly becoming fluid, borderless entities that ultimately answer to no one. Globalisation will affect key components of public health, from public hospitals and community health centres to government agencies, occupational and environmental health standards, as well as the availability and regulation of drugs and equipment. This is occurring as multinational insurance companies and managed-care organisations (MCOs) cross international borders. Since the early 1990s, institutions such as the World Bank and the International Monetary Fund have required state-owned hospitals and clinics in Third World countries to be privatised before loans are considered. Not infrequently, those formerly state-owned and -run hospitals were purchased by U.S. and European insurance companies which then introduced U.S. models of managed care, despite the vastly different history of healthcare in those countries.

Other challenges arising from the internationalisation of medicine include health tourism, and the increasing need for standardisation of medical practice. To what extent can or should the work of doctors in the various hospitals be standardised? It is dangerous to try to herd doctors into a corral made of the latest computer technology. One can understand how difficult it would be to persuade them to follow regimented patterns in their work. For instance, the transition to a computerised system for charting clinical information presents a problem to those charged with establishing a workable IT strategy. Younger doctors who are well versed in computer technology will have less difficulty accepting such a system, compared to older doctors who are not particularly computer-literate. Making the work of doctors more computer-compatible is only one factor that management has to consider. They must also accept that many doctors will resist applying sophisticated technology to their day-to-day activities. Management should not attempt to change the essential features of how doctors work in its community hospitals. That spark of independence still glows in the psyche of many doctors, and trying to regiment them will only increase its intensity [3].

There is no single correct answer to the question of “what business are we in”; instead, it will depend on our individual perspectives. Invariably one will have to manage a limited amount of resources to achieve one's aims. This is basic economics, which is a social science that studies the allocation of scarce resources used to produce goods and services that satisfy consumers' unlimited wants and needs. The perspectives change with shifts in government policies, social factors as well as commercial interests. We are always having to choose between all the different alternatives, but what is alarming is that the basis on which these decisions are made is often arbitrary and tied to whom you know in the “old boys/girls” network.

Maybe it is time to adopt a different way of thinking, namely integrative thinking which generates options and new solutions, and creates a sense of limitless possibilities. Conventional thinking wears us away with every apparent reinforcement of the lesson that life is about accepting unattractive trade-offs. Fundamentally, the conventional thinker prefers to accept the world as it is, whereas the integrative thinker welcomes the challenge of shaping the world for the better [8].
